# Cortical superficial siderosis is associated with reactive astrogliosis in cerebral amyloid angiopathy

**DOI:** 10.1186/s12974-023-02872-0

**Published:** 2023-08-27

**Authors:** Corinne A. Auger, Valentina Perosa, Steven M. Greenberg, Susanne J. van Veluw, Mariel G. Kozberg

**Affiliations:** 1 MassGeneral Institute for Neurodegenerative Disease, Massachusetts General Hospital, Harvard Medical School, 114, 16th Street, Boston, MA 02129 USA; 2 J. Philip Kistler Stroke Research Center, Massachusetts General Hospital, Harvard Medical School, 175 Cambridge Street, Boston, MA 02114 USA

**Keywords:** Cortical superficial siderosis, Cerebral amyloid angiopathy, Neuroinflammation, Reactive astrocytes, Astrogliosis

## Abstract

**Background:**

Cortical superficial siderosis (cSS) has recently emerged as one of the most important predictors of symptomatic intracerebral hemorrhage and is a risk factor for post-stroke dementia in cerebral amyloid angiopathy (CAA). However, it remains unknown whether cSS is just a marker of severe CAA pathology or may itself contribute to intracerebral hemorrhage risk and cognitive decline. cSS is a chronic manifestation of convexal subarachnoid hemorrhage and is neuropathologically characterized by iron deposits in the superficial cortical layers. We hypothesized that these iron deposits lead to local neuroinflammation, a potentially contributory pathway towards secondary tissue injury.

**Methods:**

Accordingly, we assessed the distribution of inflammatory markers in relation to cortical iron deposits in post-mortem tissue from CAA cases. Serial sections from the frontal, parietal, temporal, and occipital lobes of nineteen autopsy cases with CAA were stained with Perls’ Prussian blue (iron) and underwent immunohistochemistry against glial fibrillary acidic protein (GFAP, reactive astrocytes) and cluster of differentiation 68 (CD68, activated microglia/macrophages). Digitized sections were uploaded to the cloud-based Aiforia^®^ platform, where deep-learning algorithms were utilized to detect tissue, iron deposits, and GFAP-positive and CD68-positive cells.

**Results:**

We observed a strong local relationship between cortical iron deposits and reactive astrocytes. Like cSS-related iron, reactive astrocytes were mainly found in the most superficial layers of the cortex. Although we observed iron within both astrocytes and activated microglia/macrophages on co-stains, there was no clear local relationship between the density of microglia/macrophages and the density of iron deposits.

**Conclusion:**

Iron deposition resulting from cSS is associated with local reactive astrogliosis.

**Supplementary Information:**

The online version contains supplementary material available at 10.1186/s12974-023-02872-0.

## Background

**Fig. 1:**
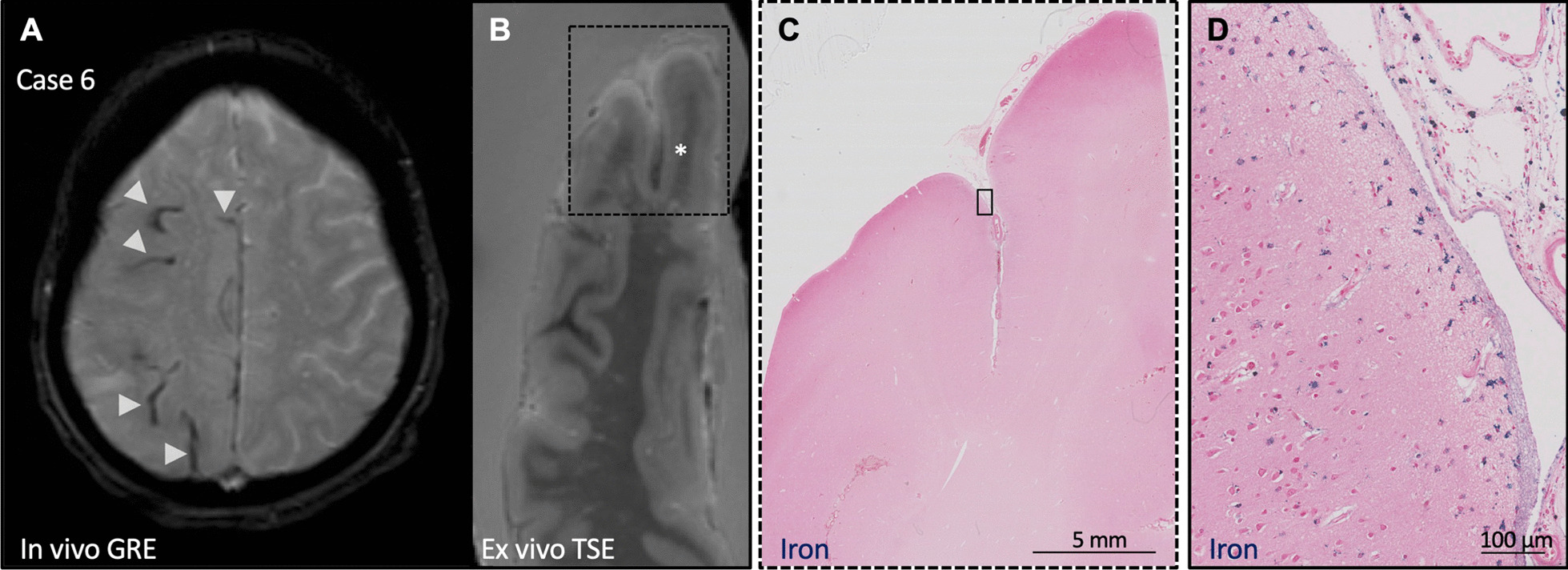
cSS on MRI and histopathology. **A** In vivo GRE MRI scan of a patient with a clinical diagnosis of probable CAA with multifocal cSS (arrowheads). **B** Ex vivo T2-weighted turbo-spin echo (TSE) MRI of one hemisphere from the same case, * indicates region of cSS. **C** Histopathological section from region outlined in B stained for iron. **D** Inset of iron stain indicated in C demonstrating iron deposits in the leptomeninges and superficial cortex

Cerebral amyloid angiopathy (CAA) is a cerebral small vessel disease histopathologically characterized by the buildup of amyloid-β (Aβ) in the walls of leptomeningeal and cortical blood vessels that leads to intracerebral hemorrhage (ICH) and cognitive impairment [1, 2]. Cortical superficial siderosis (cSS), a chronic manifestation of acute convexal subarachnoid hemorrhage, is common in patients with CAA, occurring in around 60% of histopathologically proven cases [3]. On T2*-weighted gradient echo (GRE) and susceptibility-weighted MRI sequences, cSS appears as a hypointense line tracing the edge of the cortex; on histopathology, it corresponds to iron-positive hemosiderin deposits in the subarachnoid space and superficial cortical layers (Fig. 1) [4, 5]. cSS is a hallmark of CAA and part of the Boston criteria for the condition’s possible or probable diagnosis [2, 6].

cSS is one of the most important predictors of first-time and recurrent ICH in patients with CAA [7, 8]. The incidence of ICH in patients with disseminated cSS, defined as cSS appearing in more than three sulci, is 12.5% per year, compared to 3.9% in patients with a clinical diagnosis of CAA without cSS [9, 10]. Disseminated cSS is also a risk factor for new onset of post-stroke dementia, which 10% of patients will develop within one year after ICH [11].

Prior work from our group examined the histopathological underpinnings of cSS and reported significantly higher numbers of reactive astrocytes in brains with cSS, suggesting a possible chronic inflammatory state resulting from iron deposits [4]. Studies have also shown that brains with cSS have more severe leptomeningeal CAA, less cortical CAA, and fewer cortical microbleeds than CAA brains without cSS [4, 12]. Given the relative sparing of the cortical vasculature from Aβ deposition, the mechanisms through which cSS may lead to ICH and cognitive impairment remain largely unknown. However, neuroinflammation is implicated in arteriolar remodeling, which may be a key step in generating CAA-related hemorrhage [13]. We hypothesized that secondary tissue injury related to neuroinflammation in response to cortical iron may play an important role in causing bleeding events.

We aimed to determine whether there is a local relationship between iron deposits and inflammatory cells in autopsy samples from patients with neuropathologically confirmed clinical diagnoses of CAA. We developed a pipeline for co-registering digitized histopathological sections, detecting iron deposits and inflammatory cells using deep-learning algorithms, and quantifying spatial relationships between iron and local inflammation.

## Methods

### Cases

This study included nineteen autopsy cases who were diagnosed with probable CAA during life and had their diagnosis confirmed post-mortem on pathology (Table 1) [4]. None of the patients had received anti-amyloid immunotherapies during life. Brains were received through an ongoing brain donation program through the Massachusetts General Hospital (MGH) Hemorrhagic Stroke Research Group. After brains were extracted, they were fixed in 10% formalin and the hemisphere less affected by prior ICH was selected for histopathological examination. The cases in this study represent consecutive brains received and confirmed for CAA. Approval was obtained from the MGH institutional review board (protocol number: 2021P001920) and informed consent from donors’ legal representatives prior to autopsy.

**Table 1:** Characteristics of the pathological cohort

Case No	Age at death (years)	Sex	Post-mortem interval (hours)	Cumulative cortical CAA burden on histopathology (Love score)^a^ [30]	ICH terminal event	General neuropathology findings^b^
1	80	M	Unknown	5	No	CAA (VS grade 4), A3B3C2
2	70	M	16	9	Yes	CAA (severe, widespread), arteriolosclerosis (moderate), A3B3C1
3	76	M	27	7	No	CAA (VS grade 4), arteriolosclerosis (severity not rated), A3B3C2
4	65	M	14	7	Yes	CAA (VS grade 4), A3B1C2
5	81	M	Unknown	5	No	CAA (VS grade 2), arteriolosclerosis (moderate), A3B2C2
6	70	F	Unknown	6	Yes	CAA (VS grade 3), arteriolosclerosis (mild), A3B1C1
7	67	M	Unknown	10	No	CAA (VS grade 4), arteriolosclerosis (mild), A3B3C2
8	69	M	36	10	Yes	CAA (VS grade 3), arteriolosclerosis (mild), A3B1C2
9	64	F	30	8	Yes	CAA (VS grade 3), arteriolosclerosis (mild), A3B2C3
10	79	F	37	8	Yes	CAA (VS grade 3), A3B3C2
11	67	M	24	5	No	CAA (VS grade 3), arteriolosclerosis (moderate to focally severe), A3B1C1
12	88	F	11	8	Yes	CAA (VS grade 3), A2B3C2
13	67	F	16	10	No	CAA (VS grade 3), arteriolosclerosis (moderate), A3B3C2
14	84	F	32	8	No	CAA (VS grade 3), arteriolosclerosis (severe), A3B2C2
15	67	M	Unknown	12	Yes	CAA (VS grade 4), arteriolosclerosis (severe), A3B1C2
16	76	M	20	11	No	CAA (VS grade 4), arteriolosclerosis (severe), A3B3C3
17	78	F	24	7	No	CAA (VS grade 2), arteriolosclerosis (moderate), A3B2C1, hippocampal sclerosis (TDP-43 negative)
18	86	M	20	10	No	CAA (VS grade 3), arteriolosclerosis (severe), A3B3C3, TDP-43 proteinopathy, Lewy body disease (amygdala predominant)
19	85	M	Unknown	12	No	CAA (VS grade 4), A3B3C3, arteriolosclerosis (moderate), Lewy body disease (brainstem predominant)

*M* male; *F* female; *CAA* cerebral amyloid angiopathy; *ICH* intracerebral hemorrhage

^a^CAA severity was evaluated on Aβ-stained sections from predefined regions of frontal, temporal, parietal and occipital cortices using a 4-point scale (0, absent; 1, scant Aβ deposition; 2, some circumferential Aβ; 3, widespread circumferential Aβ) following consensus criteria [30]

^b^Routine neuropathological assessments including Vonsattel score (VS) of CAA severity (not available for case 2) [31], arteriolosclerosis screen, and the National Institute on Aging-Alzheimer’s Association score for Alzheimer’s disease neuropathologic changes (ABC) [32]. Brains were also screened for Lewy bodies and hippocampal sclerosis as per [32]. These assessments were taken from routine neuropathological reports of the contralateral hemisphere of each case

### Histopathology

Formalin fixed tissue was cut into 1 cm-thick coronal slabs, and samples were taken from predefined areas of the frontal, temporal, parietal, and occipital lobes, each including cortex and juxtacortical white matter. Blocks were dehydrated, embedded in paraffin, cut into 6 μm-thick serial sections with a microtome, and mounted on slides. As outlined below, adjacent sections were stained with Perls’ Prussian blue for iron, with hematoxylin and eosin (HE), with Luxol fast blue hematoxylin and eosin (LHE), and with markers of reactive astrocytes and activated microglia/macrophages via immunohistochemistry.

To stain for iron deposits, slides were deparaffinized, rehydrated, incubated for 30 min in a 1:1 solution of 5% hydrochloric acid and 5% potassium ferrocyanide, counterstained with filtered Neutral Red solution, dehydrated, and cover slipped with Fisher Chemical Permount mounting medium. An example of an iron stain from a region of cSS observed on in vivo and ex vivo MRI is shown in Fig. 1.

For the HE stain, slides were deparaffinized, rehydrated, and incubated in hematoxylin for seven minutes, then dipped in acid alcohol to remove excess hematoxylin and incubated in eosin for three minutes. Slides were dehydrated and cover slipped.

For the LHE stain, slides were deparaffinized, rehydrated, and baked in Luxol fast blue for two hours at 60 °C. They were then rehydrated, dipped in Luxol reducer to remove excess Luxol, submerged in hematoxylin for ten minutes, dipped in acid alcohol to remove excess hematoxylin, and finally submerged in eosin for six minutes. Slides were dehydrated and cover slipped.

Brightfield immunohistochemistry was performed for glial fibrillary astrocytic protein (GFAP) (rabbit, catalog # G9269; Sigma, St. Louis, MO; 1:1,000) and cluster of differentiation 68 (CD68) (mouse, catalog # M0814; Agilent Technologies, Santa Clara, CA; 1:500). Immunohistochemistry for CD68 was carried out on a fully automated stainer (Bond Rx, Leica), while immunohistochemistry for GFAP was performed manually. For GFAP, sections were deparaffinized and rehydrated through a series of xylene and graded ethanol dilutions. Sections were incubated in 3% H_2_O_2_ to block endogenous peroxidase activity (20 min), then heated in citrate buffer for antigen retrieval (pH 6, 20 min). Tissue was blocked with normal horse (GFAP) or goat serum (CD68) (1 h), then incubated with the primary antibody overnight at 4 °C. The following day, biotinylated secondary antibody was applied (1 h), followed by a mixture of avidin and biotinylated HRP (Vectastain ABC kit, Vector laboratories, 30 min), then 3,3’-Diaminobenzidine (DAB, Vector laboratories, 3 min). Sections were briefly counterstained with hematoxylin then dehydrated and cover slipped with Permount mounting medium (Fisher Chemical).

Additional frontal lobe sections from 5 cases with the highest densities of iron deposits in iron stained frontal lobe sections (calculated as the number of Aiforia^®^-identified iron objects divided by the cortical tissue area), in the absence of macrohemorrhage, were co-stained for iron and GFAP and for iron and CD68. For co-stains, immunohistochemistry was performed following the DAB protocol detailed above, followed by incubation in a 1:1 solution of 5% hydrochloric acid and 5% potassium ferrocyanide (30 min) and a counterstain with filtered Neutral Red solution. Slides were then dehydrated and cover slipped.

### Deep learning for histopathological tissue and object detection

Stained sections were digitized with a NanoZoomer Digital Pathology-HT scanner (C9600-12; Hamamatsu Photonics, Hamamatsu, Japan), using a 20× objective. The digitized iron (Perls’ Prussian blue), GFAP, and CD68 stains were uploaded to the cloud-based Aiforia^®^ platform (www.cloud.aiforia.com; Fimmic Oy, Helsinki, Finland; v5.5) for image processing, where the cortex was manually segmented, using the adjacent LHE-stained sections as references. Methods involving Aiforia^®^ have been thoroughly described elsewhere [14]. In summary, convolutional neural networks were trained to recognize tissue and objects (iron deposits, GFAP-positive cells, or CD68-positive cells) via manual annotations (ground truth) drawn on images representing 10% of the dataset. Of note, our deep-learning model was trained to detect darkly stained GFAP-positive cells with a reactive astrocyte morphology. Additionally, the model was designed not to detect commonly observed GFAP-positive cells along the pial surface. Therefore, densities largely reflect reactive astrocytes within cortical tissue. Sections were manually reviewed and excluded if overall tissue mislabeling exceeded 5% or if object mislabeling exceeded 5% in two randomly selected regions per slide. The convolutional neural networks were then validated with annotations by three independent human validators in a subset of images (10% of the dataset) separate from the training set. For this project, the deep learning model for tissue detection on the iron sections and the deep learning models for tissue and object detection on the CD68 sections were updated from those trained for previous studies [4, 14], before being applied to the full dataset.

### Object density analysis

For each section, the digitized section, the coordinates of objects detected by Aiforia^®^, and Aiforia’s^®^ tissue detection within a manually drawn segmentation of the cortex were collected. The coordinates of objects were plotted in MATLAB as a matrix. The GFAP and CD68 sections were then co-registered to the corresponding iron section using an affine transformation followed by a non-rigid transformation. The same transformations were applied to the object matrix, and a scaling factor was applied to the number of objects represented in each pixel as necessary to ensure no change in the total number of objects after these transformations.

Cortex masks manually drawn in Aiforia^®^ were applied to the object matrices to exclude white matter from further analysis. Eight sections out of the 76 in the dataset for GFAP and five sections out of the 76 for CD68 were excluded from all analyses due to their failure to co-register. Another two sections were excluded from analyses comparing iron to GFAP and CD68 because of poor iron tissue detection. One more was excluded from analyses involving GFAP for poor GFAP-positive cell detection. Five sections were found to have evidence of macrohemorrhage, and these were analyzed separately as detailed in the results section. Note, due to issues with coregistration in one GFAP and a different CD68 section, only four of the macrohemorrhage sections are included in the macrohemorrhage analysis for GFAP or CD68. In total, 60 GFAP sections and 64 CD68 sections were included in the non-macrohemorrhage cSS analyses that involved co-registering iron and GFAP or CD68 sections.

### Layer analysis

For each section, masks were created to divide the cortex into artificial layers, each 1000 μm thick. Each section’s mask was reviewed, and a manual option was implemented for sections in which abnormally shaped tissue caused masks to be generated incorrectly. Masks for each layer were applied to the matrix of iron object counts and the co-registered matrices of GFAP-positive and CD68-positive cell counts. The density of objects in each layer was calculated. Only the first five layers’ densities were analyzed, since cortical thickness normally ranges from 1 to 4.5 mm [15, 16].

### Heat map analysis

For the heat map analysis, the cortex was divided into 500 μm * 500 μm “pixels”, and the number of objects within each pixel was calculated. Pixels with more than two thirds of their area outside the cortex were excluded. Iron was assessed along a categorical scale as follows: “very low” (0–5 iron objects), “low” (6–15), “medium” (16–25), and “high” (26 +). The analysis was repeated on four sections each for GFAP and CD68, adjacent to sections stained for iron that showed histopathological evidence of macrohemorrhage.

The associations between the total quantities of iron deposits and of GFAP-positive and CD68-positive cells within non-macrohemorrhage sections were also investigated via linear mixed effects (LME) models.

### Ring coefficient analysis

To assess the spatial extent of the association between iron and reactive astrocytes, a series of predicted GFAP heat maps based on iron density were generated for each section. These maps were compared to the actual GFAP heat maps.

First, the number of objects was calculated in three 500 μm-thick rings around each 500 μm * 500 μm pixel on iron sections, so that every pixel had four associated iron-count-per-pixel values, one for the pixel itself and one for each of the three rings extending out. The median number of objects on each GFAP and CD68 section, excluding pixels with no objects, was used to create a scaling factor, by which each pixel density value was multiplied.

$$d=adjusted\,objects\,per\,pixel = \frac{{section\,median}_{GFAP}}{{section\,median}_{iron}} * \frac{\sum\,objects\,in\,all\,pixels\,in\,ring}{pixels\,in\,ring}$$

Then, predicted GFAP heat maps were generated from these scaled iron heat maps by numerically solving for the most effective coefficient combination as follows. A matrix of all possible coefficient combinations was generated, with the coefficient on the central pixel always being greater than the coefficient on the first ring, the coefficient on the first ring being greater than that of the second, etc., and coefficients specified to intervals of 0.01. The coefficients always summed to 1.00. This analysis was performed four times, considering first just the pixel itself, then involving the pixel and the first ring, then the pixel and two rings, and finally the pixel and three rings.

For the analysis involving all three rings, the coefficient combination representing a situation in which the density of iron within a 500 μm * 500 μm pixel had an equal effect on the density of GFAP-positive cells within that pixel as the density of iron 1500–2000 μm away from that pixel would be 0.25–0.25–0.25–0.25, whereas the coefficient combination representing a situation in which the effect of iron on GFAP density were extremely local would be 1.00–0.00–0.00–0.00.

$${C}_{pixel}\ge {C}_{ring\,1}\ge \dots \ge {C}_{ring\,n}$$

$${C}_{pixel}+{C}_{ring\,1}+ \dots +{C}_{ring\,n}=1$$

The predicted GFAP or CD68 heat map for each coefficient combination was generated by summing the product of each coefficient and the corresponding ring’s mean iron count per pixel.

$${{predicted\,GFAP}_{pixel}=(C}_{pixel}*{d}_{pixel})+{(C}_{ring\,1}*{d}_{ring\,1})+ \dots +{(C}_{ring\,n}*{d}_{ring\,n})$$

For each number of rings, this process was conducted for every coefficient combination in each section. The coefficient combination that generated the predicted heat map most similar to the actual heat map was selected based on the mean of the absolute values of the difference between the predicted and actual GFAP heat maps in each pixel.

$${error}_{heat\,map}=\sum \left|{predicted\,GFAP}_{pixel}-real {GFAP}_{pixel}\right|$$

This error was normalized in each heat map to the median number of GFAP-positive cells per pixel, within pixels with GFAP, and the resulting value was termed the “residual.”

$${residual}_{heat\,map}= \,{error}_{heat\,map}* {median}_{all\,pixels\,containing\,GFAP}$$

### Statistical analysis

To test the relationship between the number of objects in 1000 μm artificial cortical layers, Friedman tests were performed and normalized with Kendall’s coefficient of concordance. *Post-hoc* pairwise comparisons were performed using Conover’s test with Benjamini–Hochberg p-value adjustments.

The relationships between the number of iron deposits and GFAP- or CD68-positive cells within 500 μm * 500 μm pixels were assessed using Skillings-Mack tests and *post-hoc* pairwise comparisons with Conover’s tests and Benjamini–Hochberg p-value adjustments. The same tests were used to compare object densities across cortical lobes.

The coefficients and residuals for the ring coefficient analysis were compared using repeated measures ANOVA tests, with pairwise comparisons via Tukey’s multiple comparisons tests.

LME models were fitted using the “lme4” package version 1.1–26 in R to assess the associations between iron deposit density and GFAP-positive cell density and CD68-positive cell density [17]. Iron deposit density was set as the independent variable, subject and cortical region (frontal, temporal, parietal, and occipital) were defined as random effects, and age at death and sex were defined as fixed effects. First, the data were tested for normality, heteroskedasticity, and the normality of residuals. The “influence.ME” package was used to detect overly influential subjects (defined as having a Cook’s distance above 4/n) for each analysis, and they were removed [18]. This resulted in removing one case from the GFAP-positive cell density analysis. A null model accounting for all effects except the dependent variable being tested (GFAP-positive cell density or CD68-positive cell density) and a second model that also accounted for the dependent variable were fitted to the data. The model fits were compared via a likelihood ratio test, their Akaike information criteria (AIC), and their Bayesian information criteria (BIC).

All statistical analyses were performed in R, version 4.1.1, except for the repeated measures ANOVAs, which were performed with GraphPad Prism version 9. Graphs were created with GraphPad Prism version 9, R version 4.1.1, and MATLAB version 2017b. A threshold of α < 0.05 was used to determine statistical significance, and all tests were two-tailed.

## Results

### Iron deposits are observed within reactive astrocytes and activated microglia

**Fig. 2:**
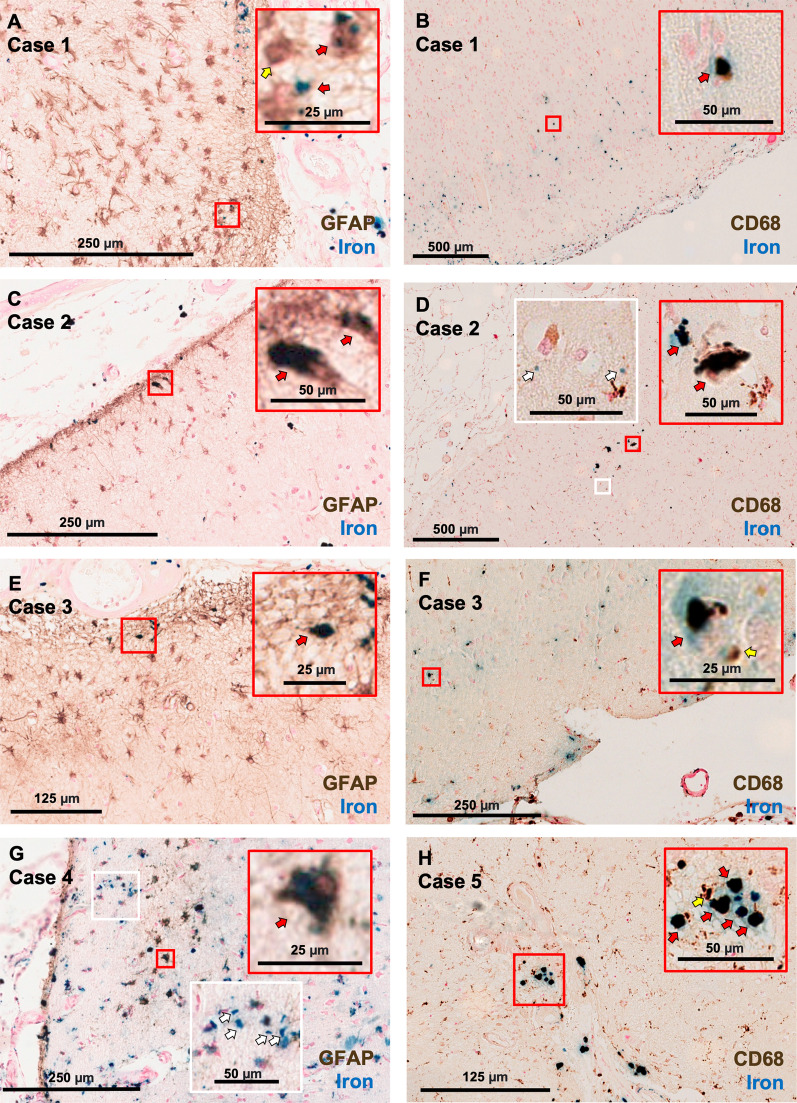
Both GFAP-positive cells and CD68-positive cells have evidence of intracellular iron. Cells positive for GFAP or CD68 appear brown, and iron deposits appear blue. The insets in red boxes contain examples of neuroinflammatory cells that co-localize with iron. Red arrows indicate examples of co-staining; yellow arrows indicate examples of GFAP- or CD68-positive cells that do not overlap with iron. The insets in white boxes contain examples of extracellular iron deposits (white arrows)

Double stains with Perls’ Prussian blue and immunohistochemistry against GFAP (n = 4) or CD68 (n = 4) were performed in sections from the frontal lobe in a subset of cases (n = 5) with the highest iron densities in Perls’ Prussian blue stained frontal lobe sections (analyzed through Aiforia^®^, see “Methods” section). The double stains revealed iron deposits colocalizing with both GFAP-positive and CD68-positive cells, suggesting phagocytosis of blood-breakdown products (Fig. 2). Iron deposits were also observed extracellularly in each double-stained section.

### Iron deposits and reactive astrocytes are predominantly observed in the superficial layers of the cortex

**Fig. 3:**
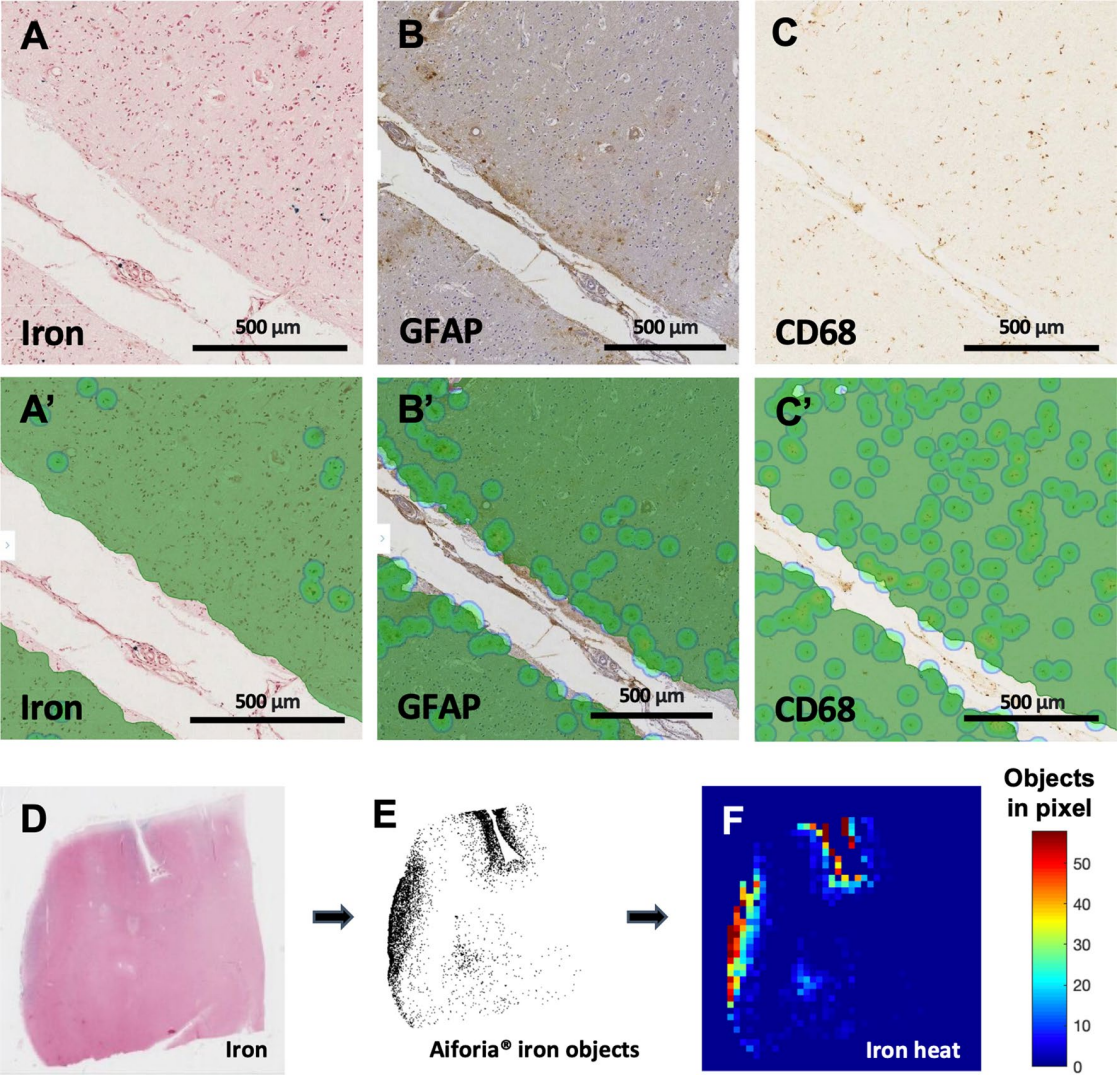
Pipeline for detecting objects with deep learning and plotting object densities. **A** Perls’ Prussian blue iron stain and immunohistochemistry against **B** GFAP and **C** CD68 in three serial 6 μm-thick sections of post-mortem human tissue. **A′–C′** Green represents Aiforia’s^®^ tissue detection. Dots represent objects (**A′** iron deposits, **B′** reactive astrocytes, **C′** activated microglia/macrophages) recognized by the three deep-learning models. **D** Example section from the temporal lobe stained for iron with Perls’ Prussian blue. **E** The coordinates of each iron deposit detected by Aiforia^®^, plotted in MATLAB. **F** The MATLAB image divided into 500 μm * 500 μm pixels with iron deposit counts plotted as a heat map

**Fig. 4:**
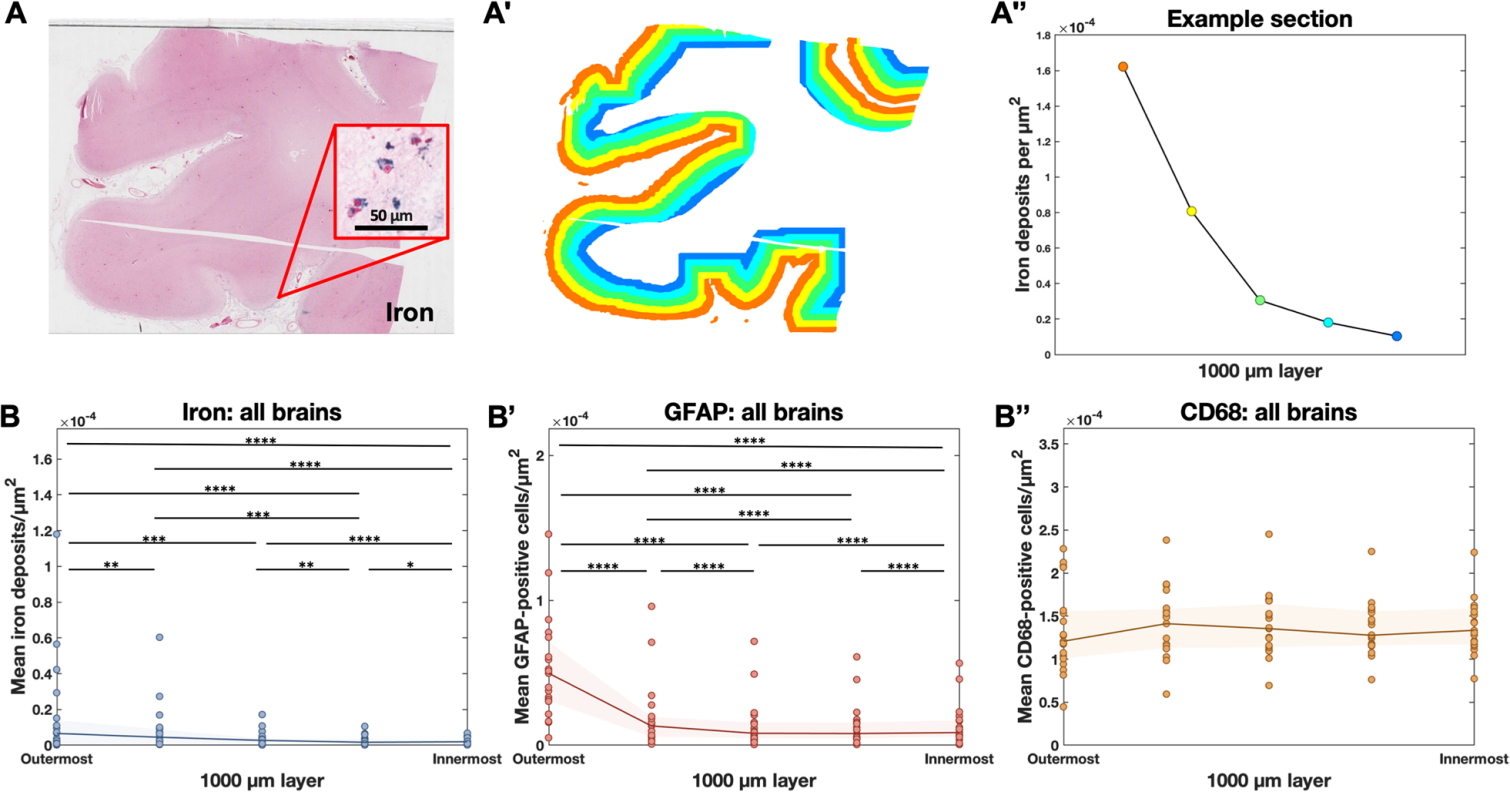
Highest densities of iron deposits and GFAP-positive cells are observed in the outermost cortical layers. **A** Example of a section from the frontal lobe stained for iron with Perls’ Prussian blue. **A′** The same section with the cortex manually segmented in Aiforia^®^ then divided into artificial 1000 μm layers using an in-house MATLAB script. **A″** The mean quantity of iron objects per square µm in each layer from the section in A, plotted. **B** Mean iron, **B′** GFAP, and **B″** CD68 densities at each 1000 μm layer. Each dot represents one brain, and the density for a brain is the mean of the densities in the sections from that brain. Each brain appears in each column. The shaded area represents the interquartile range, and only the first five artificial layers of the cortex are included. Iron deposits and reactive astrocytes are mostly present in the superficial layers of the cortex; activated microglia/macrophages do not follow the same pattern. Friedman rank sum test normalized with Kendall’s coefficient of concordance, n = 19 cases. *Post-hoc* pairwise comparisons were performed using Conover’s test for a two-way balanced complete block design with Benjamini–Hochberg p-value adjustment. *p < 0.05, **p < 0.01, ***p < 0.001, ****p < 0.0001

Iron deposits associated with MRI-visible cSS have previously been observed predominantly in the outermost cortical layers on histopathology [4]. To systematically characterize this distribution, we developed a tool to measure the density of iron deposits in 1000-μm-thick artificial cortical layers (see “Methods” section, Figs. 3, 4). The mean iron density among the four cortical lobes in each brain was highest in the outermost 1000 μm layer of the cortex and decreased with each consecutive layer inward (Fig. 4B; Friedman rank sum test, n = 19 cases, Kendall *χ*^2^ (d.f. = 4) = 23.705, p < 0.0001; pairwise comparisons with Conover’s test and Benjamini–Hochberg p-value adjustment).

The densities of GFAP-positive and CD68-positive cells were also assessed in these layers. The density of GFAP-positive cells followed a similar pattern to that of iron (Fig. 4B’; Friedman rank sum test; n = 19 cases, Kendall *χ*^2^ (d.f. = 4) = 53.179, p < 0.0001; pairwise comparisons with Conover test and Benjamini–Hochberg p-value adjustment), with the exception that the GFAP-positive density was higher in layer 5 than in layer 4, potentially due to layer 5’s proximity to the white matter (the density of GFAP-positive cells is known to be higher in the white matter than in the gray matter) [19]. The density of CD68-positive cells did not differ across cortical layers (Fig. 4B″; Friedman rank sum test, n = 19 cases, Kendall *χ*^2^ (d.f. = 4) = 53.179, p = 0.2777).

### Higher local iron density is associated with a higher local density of reactive astrocytes

**Fig. 5:**
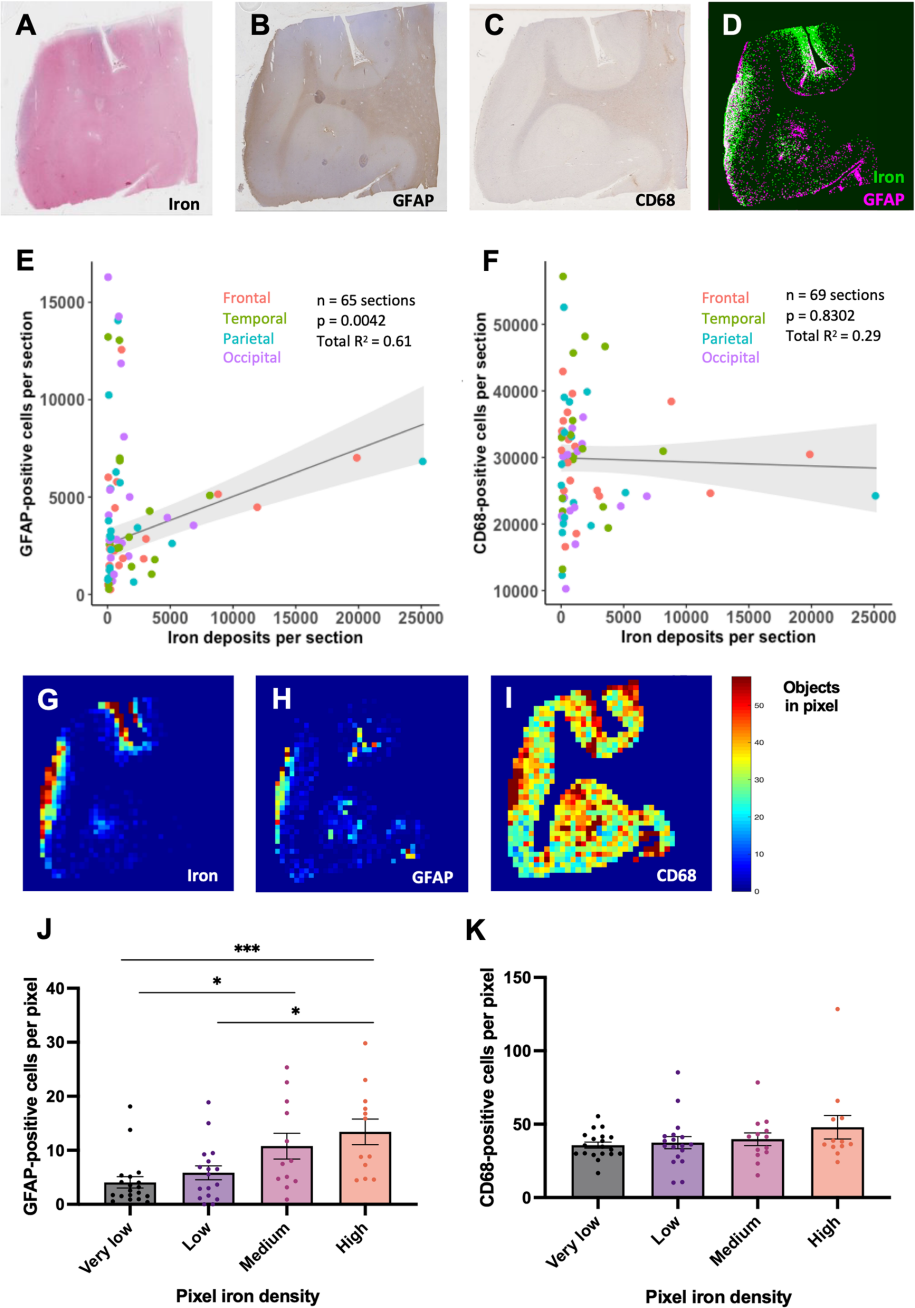
Higher densities of cortical iron deposits are locally associated with higher densities of GFAP-positive cells, and not CD68-positive cells. Examples of adjacent sections from the temporal lobe stained for **A** iron with Perls’ Prussian blue and for **B** GFAP and **C** CD68 with immunohistochemistry. **D** Overlaid plots of object detections for iron and GFAP from the same section. Iron deposits appear green; GFAP-positive cells appear pink. **E**, **F** Section-wide quantities of GFAP-positive and CD68-positive cells plotted against section-wide quantities of iron deposits with trendlines from LME models that take age at death, sex, cortical region, and subject into account. **E** There was a significant association between the number of GFAP-positive cells and the number of iron deposits within whole sections. For more details, see Table 2. **F** There was no significant association between the number of CD68-positive cells and the number of iron deposits within whole sections. For more details, see Table 3. **G**–**I** Heat maps for iron, GFAP, and CD68 with 500 μm * 500 μm pixels. **J**, **K** Plots of GFAP-positive or CD68-positive cell density vs. iron deposit density. Each dot represents one brain, and the GFAP-positive or CD68-positive cell density for a brain in one iron category is the mean of the positive cell densities in all pixels in that brain that fall within that category. Each brain may appear in each column. Skillings-Mack test, n = 19 cases. *Post-hoc* pairwise comparisons were performed using a Conover’s test with a Benjamini–Hochberg p-value adjustment. *p < 0.05, ***p < 0.001. **J** Mean GFAP-positive cell densities are higher in pixels with higher iron burden. Skillings-Mack *χ*^2^ (d.f. = 3) = 22.46, p = 5.2 E−05. **K** CD68-positive cell densities did not correlate with iron densities within pixels. Skillings-Mack *χ*^2^ (d.f. = 3) = 4.569, p = 0.2062

To assess the relationship between the density of each inflammatory marker and the density of iron deposits within sections, LME models were applied to sections sampled from each of the four cortical lobes in nineteen cases, with sex, age at death, and quantity of iron deposits as fixed effects and subject and brain region as random effects. Higher numbers of iron deposits across whole sections were associated with more GFAP-positive cells (Fig. 5E, Table 2; n = 65 sections across 18 cases, *χ*^2^ (d.f. = 1) = 8.18, p = 0.0042). Iron deposit quantity was the only significant effect (p = 0.0032) in the model with the best fit, which explained 61% of the variance in GFAP-positive cell quantity (Fig. 5E, Table 2). There was no significant association between the number of iron deposits and the number of CD68-positive cells (Fig. 5F, Table 3; n = 69 sections across 19 cases, *χ*^2^ (d.f. = 1) = 0.05, p = 0.8302).

**Table 2:** Associations between density of iron deposits and GFAP-positive cells

	Null model	Model including iron deposit density
Age at death (years)		
Estimate 95% Confidence interval p-value	51.95 [− 101.49, 204.54] p = 0.491	70.15 [− 82.38, 221.87] p = 0.352
Sex		
Estimate 95% Confidence interval p-value	1679 [− 704, 4010] p = 0.157	2106 [− 260, 4436] p = 0.080
Density of iron deposits (objects/mm^2^)		
Estimate 95% Confidence interval p-value		0.2440 [0.0806, 0.4041] **p = 0.003**
Log Likelihood	− 605.4	**− 601.3**
AIC	1223	**1217**
BIC	1236	**1232**
Total R^2^	0.49	**0.61**

Summary of the LME model explaining the density of GFAP-positive cells. Age at death, sex, and density of iron deposits were set as fixed effects; subject and cortical region were set as random effects. An estimate and confidence interval for each of the fixed effects is detailed for each model, along with statistical significance. Significant effects and overall statistics for the best model are in bold. The AIC (Akaike information criterion) and BIC (Bayesian information criterion) are measures of model fit that take model complexity into account. A lower AIC and BIC indicate a better model

**Table 3:** Associations between density of iron deposits and CD68-positive cells

	Null model	Model including iron deposit density
Age at death (years)		
Estimate 95% Confidence interval p-value	109.3 [− 267.9, 495.0] p = 0.559	105.0 [− 271.9, 492.4] p = 0.576
Sex		
Estimate 95% Confidence interval p-value	− 1857 [− 8037, 4224] p = 0.538	− 1957 [− 8161, 4195] p = 0.520
Density of iron deposits (objects/mm^2^)		
Estimate 95% Confidence interval p-value		− 0.06056 [− 0.6189, 0.5034] p = 0.829
Log Likelihood	− 724.66	**− 724.64**
AIC	**1461**	1463
BIC	**1475**	1479
Total R^2^	**0.29**	0.29

Summary of the LME model explaining the density of CD68-positive cells. Age at death, sex, and density of iron deposits were set as fixed effects; subject and cortical region were set as random effects. An estimate and confidence interval for each of the fixed effects is detailed for each model, along with statistical significance. Significant effects and overall statistics for the best model are in bold. The AIC (Akaike information criterion) and BIC (Bayesian information criterion) are measures of model fit that take model complexity into account. A lower AIC and BIC indicate a better model

To determine whether there was a local relationship between iron deposits and GFAP- and CD68-positive cells, co-registered coordinates of detected iron deposits and GFAP-positive and CD68-positive cells were converted to heat maps displaying object counts within 500 μm * 500 μm regions (termed “pixels”) (Fig. 5G–I). The mean number of GFAP-positive cells/pixel in pixels designated to each iron density category (including sections from frontal, parietal, occipital, and temporal lobes) was significantly higher in higher iron density categories (Fig. 5J; Skillings-Mack test, n = 19 cases, *χ*^2^ (d.f. = 3) = 22.46, p < 0.0001; pairwise comparisons with Conover’s test and Benjamini–Hochberg p-value adjustment; very low–medium: p = 0.0114, low–high: p = 0.0171, very low–high: p = 0.0005). The number of CD68-positive cells/pixel did not change with increasing iron density (Fig. 5K; Skillings-Mack test, n = 19 cases, *χ*^2^ (d.f. = 3) = 4.569, p = 0.2062).

The densities of iron deposits and CD68-positive cells did not differ significantly between cortical lobes (Additional file 1: Fig. S1A, 1C; Skillings-Mack test, n = 19 cases, *χ*^2^ (d.f. = 3) = 1.386, p = 0.7088 for iron, *χ*^2^ (d.f. = 3) = 4.393, p = 0.2220 for CD68). The density of GFAP-positive cells varied significantly between lobes and tended to be highest in the occipital lobe (Additional file 2: Fig. S2B; Skillings-Mack test, n = 19 cases, *χ*^2^ (d.f. = 3) = 7.962, p = 0.0468).

Astrocyte density is known to be higher in the first cortical layer than in deeper layers, as supported by the comparisons of the number of GFAP-positive cells in the outermost 1000 μm of the cortex to deeper cortical areas (Fig. 3B′) [20]. The heat map analysis was repeated including only pixels in the outermost 1000 µm of the cortex (Additional file 2: Fig. S2D–F) and again only including pixels in the deeper layers of the cortex (Additional file 2: Fig. S2J–L). Within 250 μm * 250 μm pixels for the outermost layer and 500 μm * 500 μm pixels for the rest of the cortex, we again observed significant associations between the density of iron deposits and the density of GFAP-positive cells, indicating that the two markers’ tendency to accumulate along the edge of the cortex was not the only reason for their local association (Additional file 2: Fig. S2H, M; Skillings-Mack test, n = 19 cases, *χ*^2^ (d.f. = 3) = 8.671, p = 0.0340 for edge regions, *χ*^2^ (d.f. = 3) = 19.83, p = 0.0002 for non-edge regions; pairwise comparisons with Conover's test and Benjamini–Hochberg p-value adjustment; very low–high in non-edge regions: p = 0.0101).

Sections with evidence of macrohemorrhages were analyzed separately. In the macrohemorrhage sections, the number of GFAP-positive cells per pixel was numerically higher with higher numbers of iron deposits (Additional file 3: Fig. S3F; Skillings-Mack test, n = 4 cases, *χ*^2^ (d.f. = 3) = 6.9, p = 0.0752). The number of CD68-positive cells per pixel increased significantly with higher numbers of iron deposits (Additional file 3: Fig. S3G; Skillings-Mack test, n = 4 cases, *χ*^2^ (d.f. = 3) = 8.1, p = 0.0440). Note, these sections had evidence of more recent bleeding with observed hemosiderin deposits and occasionally red blood cells.

### Most of iron deposits’ effect on reactive astrocyte density is observed within ~ 1 mm

**Fig. 6:**
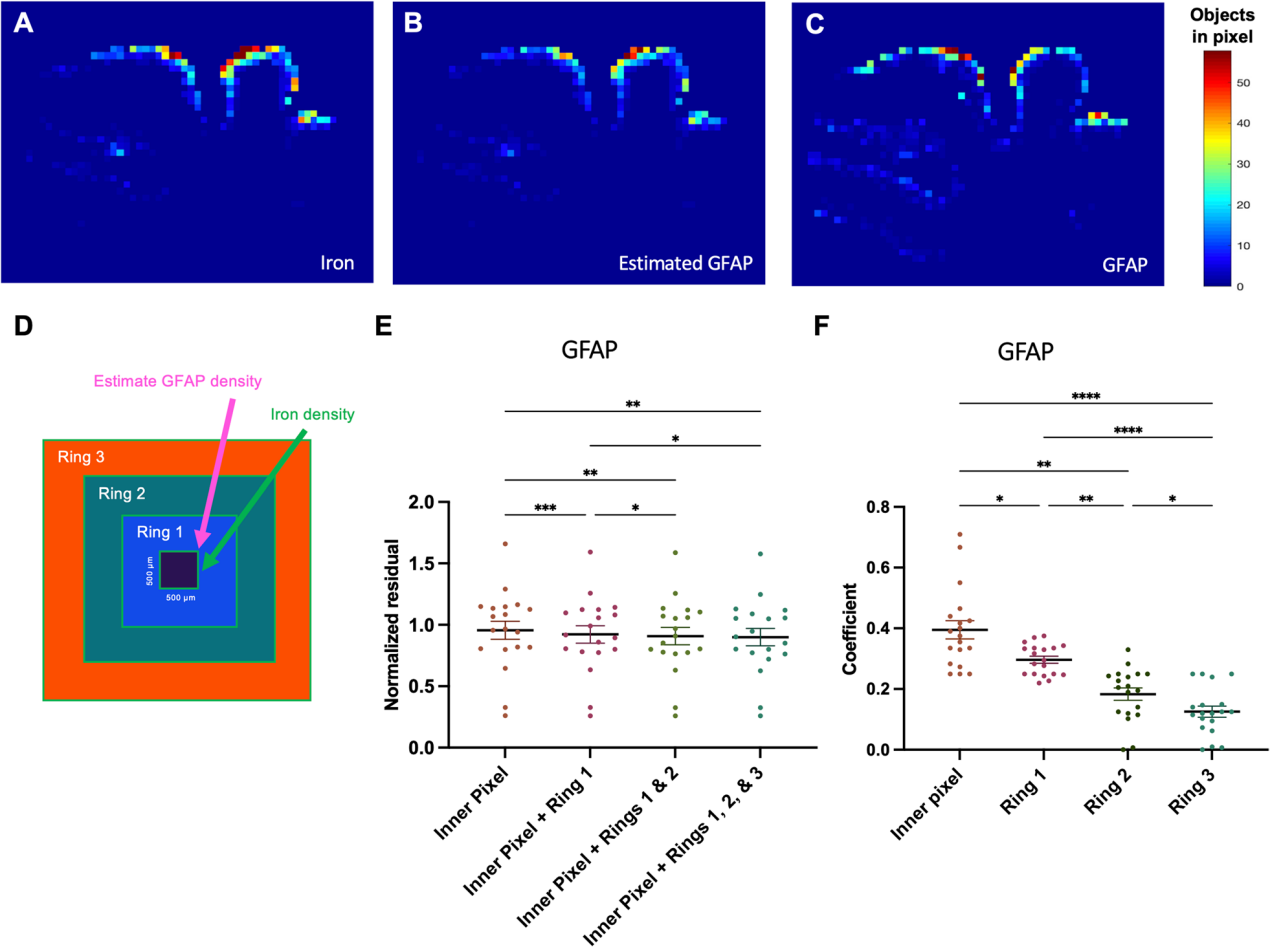
Iron heat maps can be used to predict GFAP-positive cell heat maps. **A** Example iron heat map for a section from the temporal lobe. **B** Estimated GFAP heat map based on the best coefficient combination for the iron densities in rings extending out from each pixel. **C** Actual GFAP heat map of the same section. **D** Schematic illustrating the 500 μm-thick rings extending outward from each iron pixel. **E** Residuals (comparing models with different numbers of rings surrounding the pixel) and **F** coefficients (from the model with the inner pixel pixel and all three rings) from the predicted GFAP heat maps that best matched the real heat maps. Each point represents the mean of the residuals or coefficients for sections in a brain. Repeated measures ANOVA test, n = 19 cases. *Post-hoc* pairwise comparisons were performed using Tukey’s multiple comparisons test. *p < 0.05, **p < 0.01, ***p < 0.001, ****p < 0.0001. **E** The density of GFAP-positive cells in a pixel is influenced by the density of iron deposits up to 1000 μm away (p = 0.0003). **F** The predictive effect of iron deposit density on GFAP-positive cell density decreases with distance (p < 0.0001)

To investigate the spatial extent of iron deposits’ effect on reactive astrocytes, we employed an analysis comparing combinations of weighting coefficients on the iron density values in rings extending outward from each pixel (Fig. 6). The residuals decreased overall with an increase in number of rings surrounding the inner pixel to the analysis (Fig. 6F; repeated measures ANOVA, n = 19 cases, p = 0.0003; Tukey’s multiple comparisons test). They also decreased significantly with the addition of the first ring (p = 0.0002) and second ring (p = 0.0279) to the model, indicating that the density of reactive astrocytes in a region was influenced by iron deposits up to 1000 µm away. The third ring, encompassing the region 1000–1500 µm away from the inner pixel, did not add significant predictive value although trended towards decreased residuals (p = 0.0606). In the pixel + three rings model, the coefficients that resulted in the most predictive estimated heat map decreased from the pixel to the most distant ring (Fig. 6E; repeated measures ANOVA, n = 19 cases, p < 0.0001; Tukey’s multiple comparisons test, inner pixel–ring 1: p = 0.0256, inner pixel–ring 2: p = 0.0020, inner pixel–ring 3: p < 0.0001, ring 1–ring 2: p = 0.0021, ring 1–ring 3: p < 0.0001, ring 2–ring 3: p = 0.0336).

## Discussion

Our findings demonstrate that chronic cortical iron deposits in CAA-related cSS are associated with local neuroinflammation in the form of reactive astrocytes. Neuropathological observations using double stains confirmed the presence of iron within reactive astrocytes and activated microglia/macrophages, suggesting phagocytosis of blood breakdown products. These results suggest that chronic iron deposition resulting from leptomeningeal bleeding may have damaging effects on the underlying cortical parenchyma.

The focus of this work was on resident immune cells of the brain, which we hypothesized would take up iron deposits and potentially remain activated in chronic stages. The effect of chronic iron deposits appeared to be astrocyte-predominant. One might expect more involvement of activated microglia, as the erythrophagocytic activity of microglia/macrophages is known to be crucial for the effective elimination of blood and heme [21]. An explanation might be that cSS reflects the chronic stage of leptomeningeal bleeding, which likely has a neuroinflammatory profile distinct from the acute response to brain hemorrhage. Consistent with this concept is our finding that the density of microglia/macrophages was locally associated with the density of iron deposits in areas with macrohemorrhage. The few sections with macrohemorrhages contained blood breakdown products indicative of more recent bleeding, including red blood cells and many hemosiderin deposits on HE. Of note, microglial activity may also be influenced by the presence of CAA. A recent study found that the presence of the cytokine interferon-**γ** and Aβ caused cultured microglia to become iron retentive, leading to reduced phagocytic function [22]. In the context of CAA-related cSS, the presence of Aβ and a high amount of iron deposition in the cortex has the potential to overwhelm microglia, not only causing their activation and the release of cytokines but also preventing further iron uptake.

Recent findings in ICH also suggest multiple stages of the inflammatory response post-hemorrhage. One study investigating the acute inflammatory response to ICH found that circulating neutrophils and CD14 + monocytes/macrophages in a hematoma progress through two distinct stages: the first days after hemorrhage display a glycolytic and pro-inflammatory profile, while past day 4, anti-inflammatory and anti-oxidative pathways are upregulated [23]. Similarly, a study examining brain tissue samples in parallel to serum inflammatory markers in patients with ICH found an upregulation in the innate anti-inflammatory responses several days post-ICH [24]. Although we are unable to ascertain a timeline of events from the ex vivo data presented here, it is possible that brain-resident immune cells follow a similar paradigm in response to convexal subarachnoid hemorrhage and subsequent cSS, with an acute inflammatory response (possibly driven in part by microglia) followed by a longer-term (potentially restorative) local response from astrocytes. However, this study did not differentiate between proinflammatory and anti-inflammatory immune cell activation states.

The presence of cSS is strongly associated with CAA-related ICH, and potential cortical vascular damage caused by cSS-related reactive astrogliosis could represent a pathway from cSS to ICH. Vascular remodeling including vessel wall splitting and fibrinoid necrosis has previously been shown to be associated with microhemorrhages in CAA, and recent work suggests that this remodeling may in part be mediated by perivascular inflammation particularly in the form of reactive astrocytes [25, 26, 28]. Future studies are needed to determine whether the chronic inflammatory processes associated with CAA-related cSS lead to vascular damage and could result in the rupture of associated vessels. Alternatively, cSS may not lead to ICH directly but rather the two may result from similar mechanisms. Severe leptomeningeal CAA, including leptomeningeal vascular remodeling in the form of concentric splitting of the vessel wall, is notably associated with both cSS and ICH [4, 27].

A strength of this work is that all object and tissue detection was performed using deep learning, which reduces bias compared to traditional, more subjective assessments and which increases power for statistical analysis. Deep learning models are also able to recognize cell types by shape and texture, rather than by color segmentation and size, giving them higher precision than most neuropathological software. Our pipeline for processing output from these models is a new technique for histopathological analysis that could be used with a variety of markers in the future.

There are several limitations to our work. First, CD68 is expressed by both activated microglia and macrophages, and our deep learning model was not trained to differentiate between different morphologies or activation states of microglia. Either of these differences could be a confounding factor in our results, and future studies employing techniques to classify microglial morphologies may elucidate a more specific pattern [28]. Moreover, while GFAP is a strong indicator of astrocyte activation and our GFAP model was trained to recognize reactive astrocyte morphologies, this technique is not able to delineate the degree or type of individual astrocyte activation [29]. Additionally, our sample size of 19 cases is relatively small, and all tissue examined was obtained from patients with a clinical diagnosis of CAA who volunteered for brain donation, and these patients may not be representative of the whole population of individuals with a clinical diagnosis of CAA. Whether similar associations between iron deposits and inflammation can be observed in patients without CAA but with evidence of hemorrhage from the pial surface vessels (such as in traumatic brain injury) needs to be assessed in future studies. We note that the long-term damage resulting from convexal subarachnoid hemorrhage and cSS may not be limited to the effects of iron and that other blood products may also be noxious to the tissue. Finally, studies on post-mortem tissue are inherently cross-sectional, and we are limited in determining the sequence of events leading to the observed reactive astrogliosis associated with cSS.

## Conclusion

In summary, we found that cSS-related iron deposits in patients with CAA are strongly associated with local reactive astrogliosis. This neuroinflammation may provide a mechanistic link between cSS and subsequent cognitive decline and ICH.

### Supplementary Information

**Additional file 1:** **Figure S1.** Comparisons of iron deposit densities and inflammatory cell densities across cortical regions. The density of objects does not significantly differ between cortical lobes for **(A)** iron deposits (Skillings-Mack χ^2^ (d.f. = 3) = 1.386, p = 0.709), **(B)** GFAP-positive cells (Skillings-Mack χ^2^ (d.f. = 3) = 7.962, p = 0.047), or **(C)** CD68-positive cells (Skillings-Mack χ^2^ (d.f. = 3) = 4.393, p = 0.222), n = 19 cases.

**Additional file 2:** **Figure S2. **GFAP-positive and CD68-positive cell density vs. iron density in the outer and inner portions of the cortex. Examples of adjacent sections from the frontal lobe stained **(A)** for iron with Perls’ Prussian blue and for **(B)** GFAP and **(C)** CD68 via immunohistochemistry. (**D, E, F) **Heat maps consisting of only the outermost 1000 µm of the cortex, in 250 µm * 250 µm pixels, on the same slides. (**H, I) **Plots of inflammatory cell density vs. iron deposit density in the outermost 1000 µm of the cortex. Each dot represents one brain, and the inflammatory cell density for a brain in one iron category is the mean of the inflammatory cell densities for all pixels in that section that fall within that category. Each brain may appear in each column. Skillings-Mack test, n = 19 cases. *Post-hoc *pairwise comparisons were performed using Conover tests with Benjamini-Hochberg p-value adjustment. (**H)** In the outer edge of the cortex, mean GFAP-positive cell densities were higher in pixels with higher iron burden in four predefined categories. None of the pairwise comparisons were significant. (**I) **Mean CD68-positive cell densities were not significantly higher in pixels with higher iron burden. (**J, K, L)** Heat maps of the cortex without the outermost 1000 µm, in 500 µm * 500 µm pixels, on the same slides. (**M, N) **Plots of inflammatory cell density vs. iron deposit density in the cortex without the outermost 1000 µm. *p < 0.05. (**M)** In the cortex without the outer edge, mean GFAP-positive cell densities were higher in pixels with higher iron burden in four predefined categories. (**N) **In the cortex without the outer edge, mean CD68-positive cell densities were not significantly higher in pixels with higher iron burden.

**Additional file 3:** **Figure S3.** GFAP-positive and CD68-positive cell density vs. iron density in sections with macrohemorrhages. **(A)** Example section from the temporal lobe in an area with macrohemorrhage, stained with hematoxylin and eosin. (**B, C, D, E)** Serial sections from the inset region in the slide above, stained with **(B)** hematoxylin and eosin to visualize hemosiderin deposits (in hemorrhagic region indicated by red asterisk), **(C) **Perls’ Prussian blue, and immunohistochemistry against **(D)** GFAP and **(E)** CD68. Examples of GFAP-positive and CD68-positive cells are indicated by yellow arrows. (**F, G)** Reactive astrocytes and activated microglia/macrophages are present in the area around blood breakdown products. Plots of inflammatory cell density vs. iron deposit density in sections with macrohemorrhage. Each dot represents one section, and the inflammatory cell density for a section in one iron category is the mean of the inflammatory cell densities for all pixels in that brain that fall within that category. Each section may appear in each column. (**F)** In sections with a macrohemorrhage, mean GFAP-positive cell densities trended toward being higher in pixels with higher iron burden, but there was not a significant association (Skillings-Mack χ^2^ (d.f. = 3) = 6.9, p = 0.0752). (**G)** In sections with a macrohemorrhage, mean CD68-positive cell densities were higher in pixels with higher iron burden in four predefined categories (Skillings-Mack χ^2^ (d.f. = 3) = 8.1, p = 0.0440).

## Data Availability

Histopathological data are available from the corresponding author upon reasonable request. The code supporting the conclusions of this article is available in the cSS-reactive-astrogliosis repository, (https://github.com/CorinneAuger/cSS-reactive-astrogliosis).

## Abbreviations

cSS: Cortical superficial siderosis
CAA: Cerebral amyloid angiopathy
GFAP: Glial fibrillary acidic protein
CD68: Cluster of differentiation 68
Aβ: Amyloid-β
ICH: Intracerebral hemorrhage
HE: Hematoxylin and eosin
LHE: Luxol fast blue hematoxylin and eosin
DAB: 3,3′-Diaminobenzidine
