# Supplementary material for: Cortical superficial siderosis is associated with reactive astrogliosis in cerebral amyloid angiopathy
Source: J Neuroinflammation. 2023 Aug 27;20:195. doi: 10.1186/s12974-023-02872-0 (PMC10463916; doi:10.1186/s12974-023-02872-0)
Supplement: Supplementary file 1 — Additional file 1: Figure S1. Comparisons of iron deposit densities and inflammatory cell densities across cortical regions. The density of objects does not significantly differ between cortical lobes for (A) iron deposits (Skillings-Mack χ2 (d.f. = 3) = 1.386, p = 0.709), (B) GFAP-positive cells (Skillings-Mack χ2 (d.f. = 3) = 7.962, p = 0.047), or (C) CD68-positive cells (Skillings-Mack χ2 (d.f. = 3) = 4.393, p = 0.222), n = 19 cases. [file 12974_2023_2872_MOESM1_ESM.docx]

**
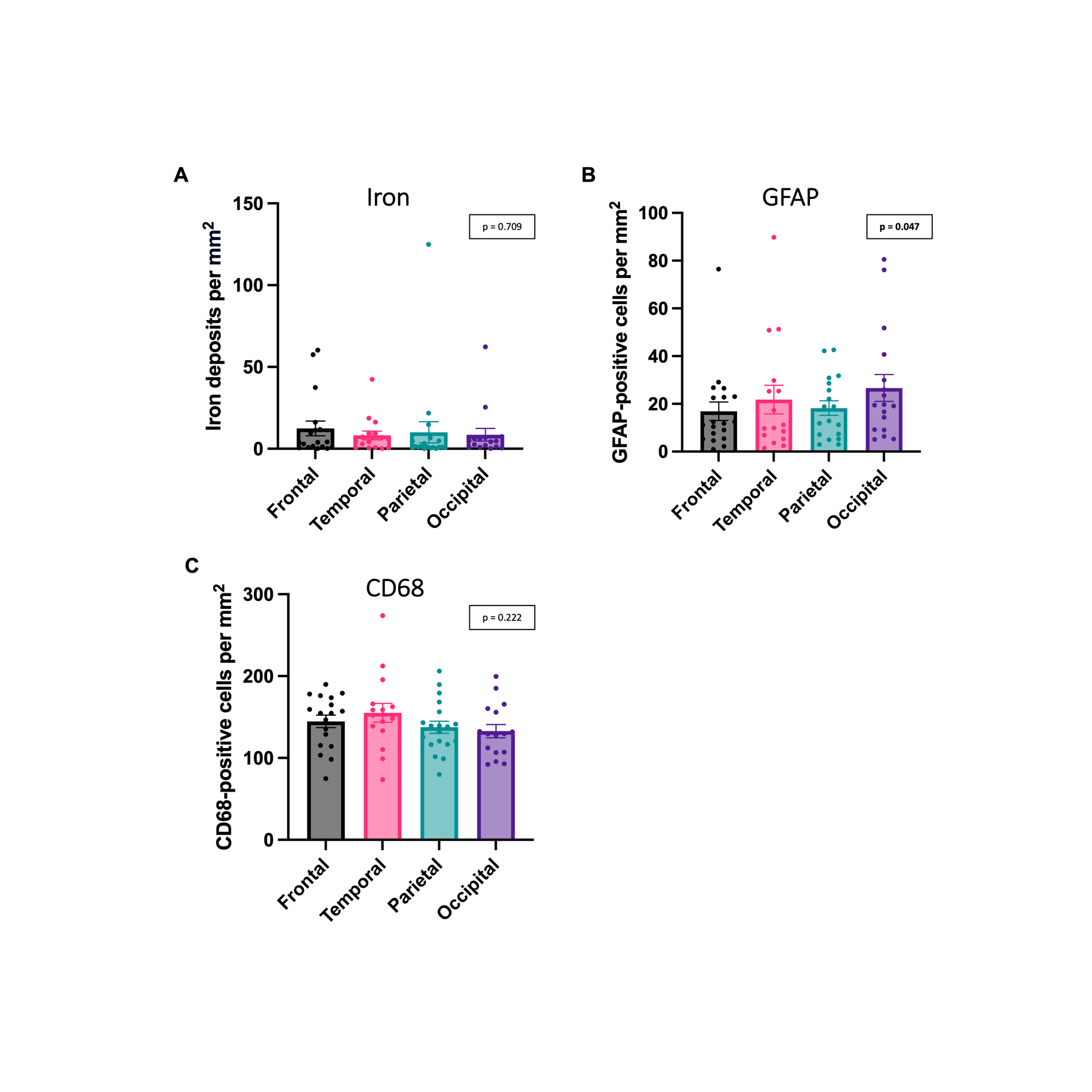
**

**Fig. S1**

**Comparisons of iron deposit densities and inflammatory cell densities across cortical regions.**

The density of objects does not significantly differ between cortical lobes for **(A)** iron deposits (Skillings-Mack χ^2^ (d.f. = 3) = 1.386, p = 0.709), **(B)** GFAP-positive cells (Skillings-Mack χ^2^ (d.f. = 3) = 7.962, p = 0.047), or **(C)** CD68-positive cells (Skillings-Mack χ^2^ (d.f. = 3) = 4.393, p = 0.222), n = 19 cases.
