# Supplementary material for: Cortical superficial siderosis is associated with reactive astrogliosis in cerebral amyloid angiopathy
Source: J Neuroinflammation. 2023 Aug 27;20:195. doi: 10.1186/s12974-023-02872-0 (PMC10463916; doi:10.1186/s12974-023-02872-0)
Supplement: Supplementary file 2 — Additional file 2: Figure S2. GFAP-positive and CD68-positive cell density vs. iron density in the outer and inner portions of the cortex. Examples of adjacent sections from the frontal lobe stained (A) for iron with Perls’ Prussian blue and for (B) GFAP and (C) CD68 via immunohistochemistry. (D, E, F) Heat maps consisting of only the outermost 1000 µm of the cortex, in 250 µm * 250 µm pixels, on the same slides. (H, I) Plots of inflammatory cell density vs. iron deposit density in the outermost 1000 µm of the cortex. Each dot represents one brain, and the inflammatory cell density for a brain in one iron category is the mean of the inflammatory cell densities for all pixels in that section that fall within that category. Each brain may appear in each column. Skillings-Mack test, n = 19 cases. Post-hoc pairwise comparisons were performed using Conover tests with Benjamini-Hochberg p-value adjustment. (H) In the outer edge of the cortex, mean GFAP-positive cell densities were higher in pixels with higher iron burden in four predefined categories. None of the pairwise comparisons were significant. (I) Mean CD68-positive cell densities were not significantly higher in pixels with higher iron burden. (J, K, L) Heat maps of the cortex without the outermost 1000 µm, in 500 µm * 500 µm pixels, on the same slides. (M, N) Plots of inflammatory cell density vs. iron deposit density in the cortex without the outermost 1000 µm. *p < 0.05. (M) In the cortex without the outer edge, mean GFAP-positive cell densities were higher in pixels with higher iron burden in four predefined categories. (N) In the cortex without the outer edge, mean CD68-positive cell densities were not significantly higher in pixels with higher iron burden. [file 12974_2023_2872_MOESM2_ESM.docx]

**
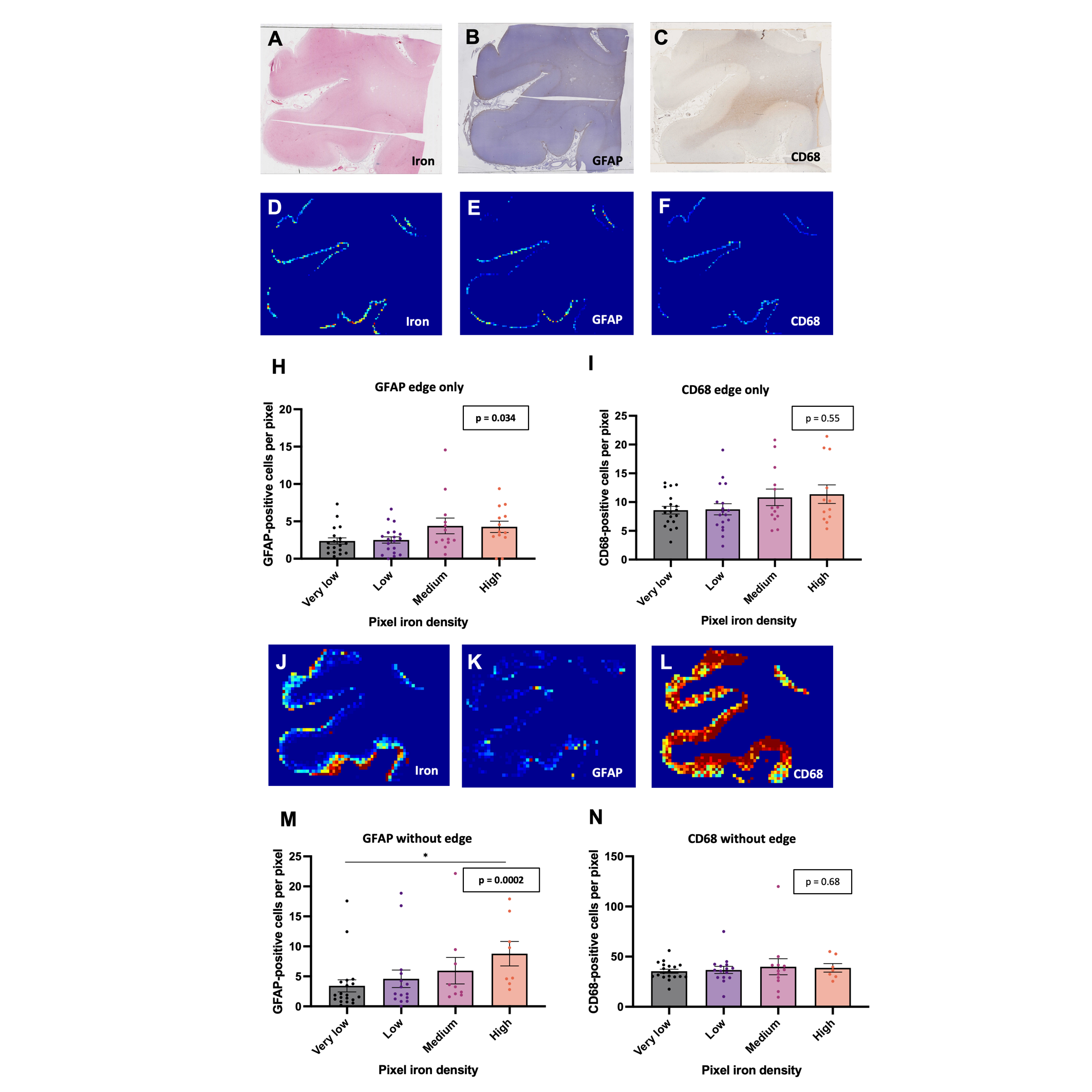
**

**Fig. S2**

**GFAP-positive and CD68-positive cell density vs. iron density in the outer and inner portions of the cortex**

Examples of adjacent sections from the frontal lobe stained **(A)** for iron with Perls’ Prussian blue and for **(B)** GFAP and **(C)** CD68 via immunohistochemistry. (**D, E, F)** Heat maps consisting of only the outermost 1000 µm of the cortex, in 250 µm * 250 µm pixels, on the same slides. (**H, I)** Plots of inflammatory cell density vs. iron deposit density in the outermost 1000 µm of the cortex. Each dot represents one brain, and the inflammatory cell density for a brain in one iron category is the mean of the inflammatory cell densities for all pixels in that section that fall within that category. Each brain may appear in each column. Skillings-Mack test, n = 19 cases. *Post-hoc* pairwise comparisons were performed using Conover tests with Benjamini-Hochberg p-value adjustment. (**H)** In the outer edge of the cortex, mean GFAP-positive cell densities were higher in pixels with higher iron burden in four predefined categories. None of the pairwise comparisons were significant. (**I)** Mean CD68-positive cell densities were not significantly higher in pixels with higher iron burden. (**J, K, L)** Heat maps of the cortex without the outermost 1000 µm, in 500 µm * 500 µm pixels, on the same slides. (**M, N)** Plots of inflammatory cell density vs. iron deposit density in the cortex without the outermost 1000 µm. *p < 0.05. (**M)** In the cortex without the outer edge, mean GFAP-positive cell densities were higher in pixels with higher iron burden in four predefined categories. (**N)** In the cortex without the outer edge, mean CD68-positive cell densities were not significantly higher in pixels with higher iron burden
