# Supplementary material for: Cortical superficial siderosis is associated with reactive astrogliosis in cerebral amyloid angiopathy
Source: J Neuroinflammation. 2023 Aug 27;20:195. doi: 10.1186/s12974-023-02872-0 (PMC10463916; doi:10.1186/s12974-023-02872-0)
Supplement: Supplementary file 3 — Additional file 3: Figure S3. GFAP-positive and CD68-positive cell density vs. iron density in sections with macrohemorrhages. (A) Example section from the temporal lobe in an area with macrohemorrhage, stained with hematoxylin and eosin. (B, C, D, E) Serial sections from the inset region in the slide above, stained with (B) hematoxylin and eosin to visualize hemosiderin deposits (in hemorrhagic region indicated by red asterisk), (C) Perls’ Prussian blue, and immunohistochemistry against (D) GFAP and (E) CD68. Examples of GFAP-positive and CD68-positive cells are indicated by yellow arrows. (F, G) Reactive astrocytes and activated microglia/macrophages are present in the area around blood breakdown products. Plots of inflammatory cell density vs. iron deposit density in sections with macrohemorrhage. Each dot represents one section, and the inflammatory cell density for a section in one iron category is the mean of the inflammatory cell densities for all pixels in that brain that fall within that category. Each section may appear in each column. (F) In sections with a macrohemorrhage, mean GFAP-positive cell densities trended toward being higher in pixels with higher iron burden, but there was not a significant association (Skillings-Mack χ2 (d.f. = 3) = 6.9, p = 0.0752). (G) In sections with a macrohemorrhage, mean CD68-positive cell densities were higher in pixels with higher iron burden in four predefined categories (Skillings-Mack χ2 (d.f. = 3) = 8.1, p = 0.0440). [file 12974_2023_2872_MOESM3_ESM.docx]

**
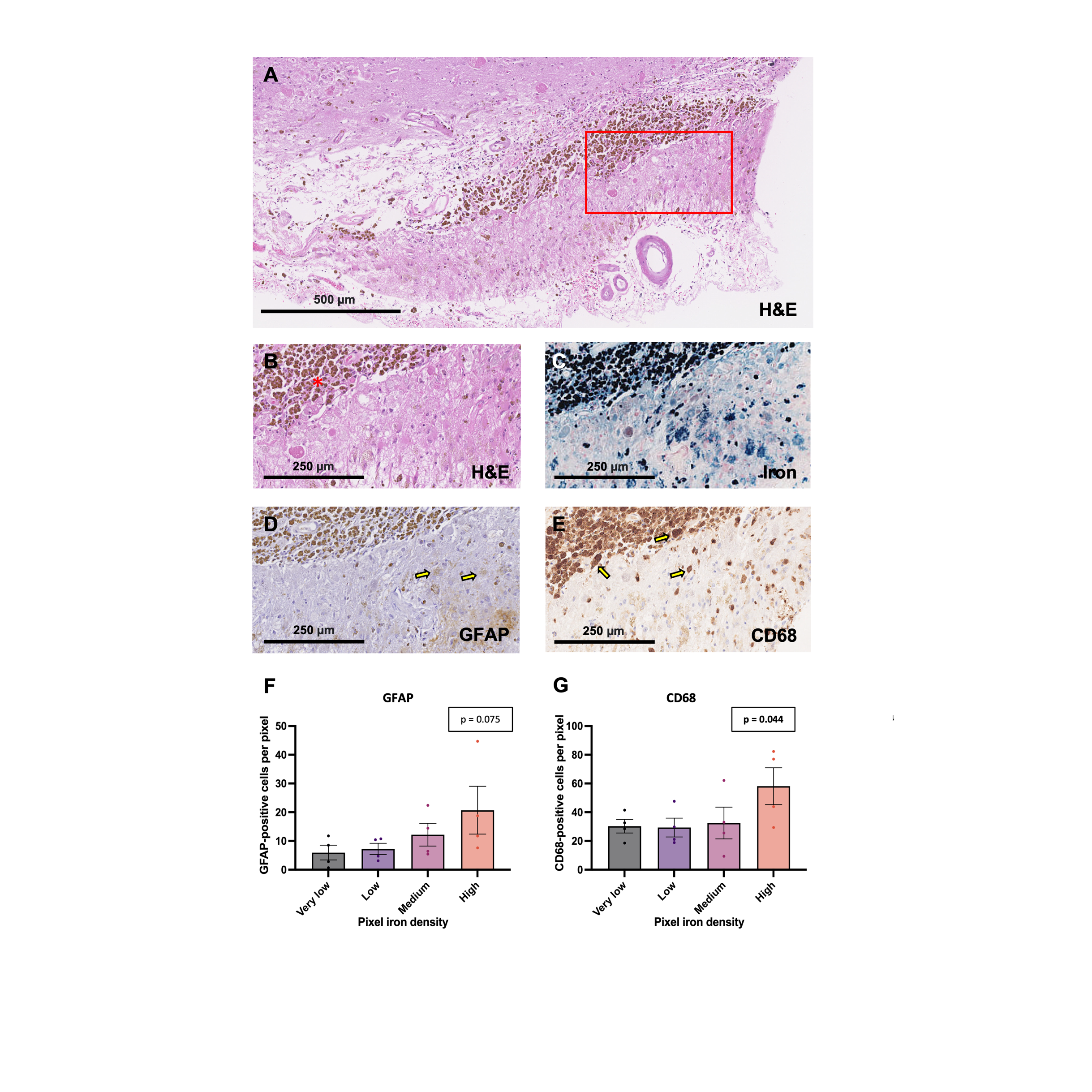
**

**Fig. S3**

**GFAP-positive and CD68-positive cell density vs. iron density in sections with macrohemorrhages.**

**(A)** Example section from the temporal lobe in an area with macrohemorrhage, stained with hematoxylin and eosin. (**B, C, D, E)** Serial sections from the inset region in the slide above, stained with **(B)** hematoxylin and eosin to visualize hemosiderin deposits (in hemorrhagic region indicated by red asterisk), **(C)** Perls’ Prussian blue, and immunohistochemistry against **(D)** GFAP and **(E)** CD68. Examples of GFAP-positive and CD68-positive cells are indicated by yellow arrows. (**F, G)** Reactive astrocytes and activated microglia/macrophages are present in the area around blood breakdown products. Plots of inflammatory cell density vs. iron deposit density in sections with macrohemorrhage. Each dot represents one section, and the inflammatory cell density for a section in one iron category is the mean of the inflammatory cell densities for all pixels in that brain that fall within that category. Each section may appear in each column. (**F)** In sections with a macrohemorrhage, mean GFAP-positive cell densities trended toward being higher in pixels with higher iron burden, but there was not a significant association (Skillings-Mack χ^2^ (d.f. = 3) = 6.9, p = 0.0752). (**G)** In sections with a macrohemorrhage, mean CD68-positive cell densities were higher in pixels with higher iron burden in four predefined categories (Skillings-Mack χ^2^ (d.f. = 3) = 8.1, p = 0.0440).
